# Medical educational culture: introducing patients to applicants as part of the medical school interview: feasibility and initial impact show and tell

**DOI:** 10.3402/meo.v21.31760

**Published:** 2016-08-11

**Authors:** Shireen Madani Sims, James W. Lynch

**Affiliations:** 1 Department of Obstetrics and Gynecology, University of Florida College of Medicine, Gainesville, FL, USA; 2 Department of Medicine, University of Florida College of Medicine, Gainesville, FL, USA

**Keywords:** patient-centered care, holistic review, medical admissions

## Abstract

**Introduction:**

The College of Medicine at our institution underwent a major curricular revision in order to develop a patient-centered context for learning. The admission process was revised to reflect this change, adopting a holistic review process, with the hope of attracting students who were particularly well suited to a patient-centered curriculum and learning culture.

**Methods:**

Patients from a single practitioner, who were accustomed to working with medical students, were asked if they would like to select the next generation of physicians. The patient's experience included a brief didactic presentation related to the patient's diagnosis and treatment. This was followed by an informal session with the applicants and the physician, where they shared their story in a small group setting. They were encouraged to share their experiences with the healthcare system, both positive and negative. The goal was to allow applicants to glean the importance of the human aspects of disease in our institutional culture of learning.

**Results:**

The response and experience were overwhelmingly positive for the patients who donated their time to participate and for our applicants. Follow-up surveys indicated that our applicants found the experience to be unique and positive. Many of the students who chose to attend our university cited the interview experience and learning culture as factors that influenced their choice of medical schools. In addition, the Liaison Committee on Medical Education cited the favorability of the admission process in their recent site visit.

**Discussion:**

Now in its fifth year, we can say that the inclusion of patients as part of the interview day is feasible as part of our admission process. We continue to make changes and monitor our progress, and we have added several other faculty members and specialties in order to ensure the program is sustainable.

Patient-centered care has become an increasing directive to the medical community, since the Institute of Medicine identified it as one of the keys to improving quality of care (1). Towards this end, the University of Florida College of Medicine (UFCOM) undertook a curriculum revision using patients’ stories to create an educational context for organizing complex material and prepare learners for a patient-centered approach in their clinical interactions. At the same time, the medical school admission office adopted a holistic review process that evaluated each applicant in a broader context of life experiences, with the hope of attracting students who were particularly well suited to a patient-centered curriculum. Including patients as part of the admission process, particularly introducing applicants to a patient on the interview day, seemed a natural extension of our desire to provide patient-centered education and care.

## Methods

The decision to include patients as part of the interview process was carefully considered, discussed, and approved by the UFCOM senior administration. It was determined by senior leadership that no ethics or IRB approval was needed. One of the authors is a practicing medical oncologist, and the first stage of this project was limited to patients from his practice. Patients were invited to participate on the interview day as an extension of their normal clinical experience. These patients were accustomed to interacting with students in clinic and have long been part of the didactic lectures in the first 2 years, volunteering to speak to students about the human side of illness. Patients thought to be appropriate were invited to participate by asking if they would like the opportunity to help pick the next generation of doctors. The response was overwhelming, with more patients interested in participating than there were on the interview days.

The interview day began with a discussion of the theory behind the patient-centered curriculum as a natural bridge to the goal of patient-centered care. To ensure an understanding of patient privacy, applicants read and signed the Health Insurance Portability and Accountability Act of 1996 (HIPAA) Privacy, Security and Breach Notification Rules form as part of their initial welcome. The patient's experience included a brief didactic presentation related to the patient's illness and treatment. This was followed by an informal session where the physician sat down with the patient and asked them to share their story in a small group setting with 10–12 applicants. The patients were asked to give a brief narration of their life and how they came to medical attention. They were encouraged to share both the positive and negative experiences with the healthcare system. Discussions covered a wide variety of topics, including presenting symptoms, experiences with treatments, the communication skills of their providers, the impact on their family and friends, and spiritual issues, all at the patient's discretion. After the applicants were given the opportunity to ask questions, the patients gave the last word offering their view of the most important characteristics of a physician. The goal was to allow applicants to glean the importance of the human aspects of disease in our institutional culture. The remainder of the interview day included a discussion of the importance of diversity, a session with our anesthesia simulator, and lunch with our current students, as well as two individual semi-scripted interviews with members of our interview committee. The day concluded with a reassembly of the interviewees and closing comments from the admission dean.

## Results

The experience was well received by both patients and applicants. Fourteen patients participated in the first year as a pilot and feasibility study, each donating approximately 3 h of their time. The patients all enjoyed the experience and said they would welcome the opportunity to participate in the future; and in fact, several have participated on more than one occasion. Patients were also invited to our reception for accepted students, and many applicants were able to reconnect with the patient from their interview day.

**Fig. 1:**
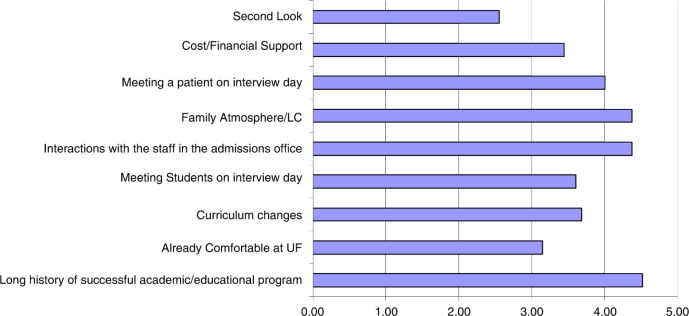
Matriculation survey from UFCOM students with our new interview format. (Students’ decision to come to UFCOM was influenced in varying degrees by the factors depicted in the bar diagram; 0 being not at all and 5 being very important – Five-point scale.)

There were 375 applicants interviewed in the first year of this program. Our post-interview survey identified meeting a patient as a unique highlight of the day. Furthermore, a fully anonymized matriculation survey asking applicants why they chose our institution showed that for many, meeting a patient during the interview was an important factor in their decision. This survey was administered during orientation to medical school via a web-based survey platform. There were 86 responses out of 135 students in a class. The students were asked to rate the importance of these factors on a Likert scale, with 5 being very important and 1 being not important at all. Figure 1 demonstrates that students considered meeting a patient on the interview day to be one of the most important factors that influenced their decision to attend the UFCOM, along with the family friendly atmosphere, the quality of the admission staff, and the excellent reputation of our institution (Fig. 1). Among verbal comments related to why students chose our institution, 75% were related to the interview day experience and the culture at our institution. Specific comments highlighted the current medical student's positive impression of their school, changes in the curriculum, friendliness of the admission staff, and meeting a patient on the interview day.

During the patient interaction, admissions’ staff members were able to observe the manner in which applicants responded to the experience, although this was not necessarily the intent. Some students were so natural in their interaction with patients that they stood out in a favorable way. On the other hand, there were also a small number of applicants who appeared quite disinterested, or during their interviews expressed a negative reaction to the encounter (e.g., ‘it was a complete waste of time’). A number of examples could be chosen to illustrate the positive impression upon applicants. However, one probably summarizes the spirit of the verbal comments.

I was fortunate to interview at my dream schools, where they were quick to highlight their Nobel laureates, clinical rankings, research accolades, cutting-edge technologies and vast resources. But … at UF the Dean of Admissions … personally introduced me to potential mentors, current students, and even a patient. UF made it abundantly clear to me that they teach their physicians to be more than competent physicians, but also compassionate, socially-conscious, decent people. Ultimately, I chose UF because their values are aligned with my own.

In the subsequent 4 years, the program has expanded to include eight other faculty members from the admission committee representing a variety of disciplines, including internal medicine, pediatrics, family medicine, and obstetrics and gynecology. Now with 5 years of experience, it has become a standard part of the UFCOM interview experience. During the semi-structured interview, interviewers ask each applicant about their impressions of the patient interaction and its purpose within our process.

In February 2015, the UFCOM had its Liaison Committee on Medical Education (LCME) site visit, and the committee discussed this program with both students and members of the admission committee. As part of our final review, the committee reported the following:

The admissions process is widely praised by students for its welcoming approach and for the unique inclusion of patients as part of the interview. Students report that this concrete demonstration of patient-centeredness was a strong factor in their choice of the University of Florida College of Medicine.

## Discussion

Now with many years of experience, the AAMC holistic admission process has been adopted by many medical schools including the UFCOM. The intent is to widen the ‘lens through which we view applicants’ (2) towards the end of selecting student physicians who will meet the healthcare needs of the future (3, 4). Such physicians not only should be ready to address the needs of underserved communities but also be motivated by compassion and a desire to serve others. The question of how to accomplish this has been the subject of much discussion. Many admission committees emphasize things such as communication skills, extracurricular activities, knowledge of the profession, and psychometrics among many others (5). Interviews have largely focused on assessing communication and social skills after applicants have been screened according to traditional metrics as well as their attributes and experiences (6). However, the validity and reproducibility of these measures have been called into question (7). The suggestion that admission processes should be judged by these criteria are predicated upon a clinical trial model and the assumption that all important attributes in future physicians are measureable. We would argue instead that many critical attributes of a physician such as work ethic, other-centeredness, integrity, and compassion, to name just a few, are by their very nature resistant to modern measurement tools. If this is the case, attempting to evaluate these sorts of attributes needs to be a part of the admission process, as imperfect as those evaluations may be.

Like many other schools, our interview day involves an attempt to demonstrate for applicants the culture of our institution. As the consensus of the faculty and leadership was to press deeper into patient-centered care, creating a patient-centered curriculum was one component of that strategy. The idea of the patient-centered interview day emerged in the wake of these changes. The patients who participated had a wide variety of characteristics, diagnoses, outcomes, and experiences with the healthcare system. Often what they had in common was a desire to be involved with the process of selecting the future generation of doctors, and they also admittedly had a warm and open relationship with their treating physicians. The doctor–patient interaction was a highlight of the experience. There existed several common themes during the meetings between the patient, treating physician, and the applicants: 1) the importance of listening to patients and communicating compassion as well as, appropriate medical information, 2) how harmful or hurtful it is to patients when we as physicians are careless or callous in our dealings with patients, 3) the critical nature of trust in the physician–patient relationship, 4) the stress on the spouse or caregiver for a sick patient, 5) the two sides of technology in medicine, with great medical advances, but the tendency for technology to impact communication between patients and their physicians.

While many medical schools have included patients as part of their process for evaluating applicants, to our knowledge this is the first attempt to have all applicants hear from a patient as part of their interview experience. Our preliminary analysis suggests that this component of our culture was an influential factor for applicants that spoke to our patient-centered philosophy. In collaboration with colleagues from our medical education office, we have developed an IRB-approved focus group based study to more formally evaluate the impact of this program. Using qualitative methods including recorded interviews with randomly selected groups of students from multiple classes, we expect to offer a more systematic evaluation in the near future.

## Conclusions

The inclusion of patients as part of the interview day is feasible and sustainable as a part of our UFCOM admission process. Moreover, many applicants with multiple acceptances identified meeting a patient as an important component of their decision to attend UFCOM both as measured by surveys and as part of our LCME review committee. A more formal evaluation will be forthcoming when our current study is completed and analyzed.
